# Berberine and Obatoclax Inhibit SARS-Cov-2 Replication in Primary Human Nasal Epithelial Cells In Vitro

**DOI:** 10.3390/v13020282

**Published:** 2021-02-11

**Authors:** Finny S. Varghese, Esther van Woudenbergh, Gijs J. Overheul, Marc J. Eleveld, Lisa Kurver, Niels van Heerbeek, Arjan van Laarhoven, Pascal Miesen, Gerco den Hartog, Marien I. de Jonge, Ronald P. van Rij

**Affiliations:** 1 Department of Medical Microbiology, Radboud Institute for Molecular Life Sciences, Radboud University Medical Center, 6500 HB Nijmegen, The Netherlands; finny.varghese@radboudumc.nl (F.S.V.); gijs.overheul@radboudumc.nl (G.J.O.); pascal.miesen@radboudumc.nl (P.M.); 2 Section Paediatric Infectious Diseases, Laboratory of Medical Immunology, Radboud Institute for Molecular Life Sciences, Radboud University Medical Center, 6500 HB Nijmegen, The Netherlands; esther.van.woudenbergh@rivm.nl (E.v.W.); Marc.Eleveld@radboudumc.nl (M.J.E.); marien.dejonge@radboudumc.nl (M.I.d.J.); 3 Centre for Immunology of Infectious Diseases and Vaccines, National Institute for Public Health and the Environment, 3721 MA Bilthoven, The Netherlands; Gerco.den.hartog@rivm.nl; 4 Department of Internal Medicine, Radboud University Medical Center, 6500 HB Nijmegen, The Netherlands; Lisa.Kurver@radboudumc.nl (L.K.); arjan.vanlaarhoven@radboudumc.nl (A.v.L.); 5 Department of Otolaryngology, Head and Neck Surgery, Radboudumc, 6500 HB Nijmegen, The Netherlands; niels.vanheerbeek@radboudumc.nl

**Keywords:** coronavirus, COVID-19, antiviral compounds

## Abstract

Severe acute respiratory syndrome coronavirus 2 (SARS-CoV-2) emerged as a new human pathogen in late 2019 and it has infected over 100 million people in less than a year. There is a clear need for effective antiviral drugs to complement current preventive measures, including vaccines. In this study, we demonstrate that berberine and obatoclax, two broad-spectrum antiviral compounds, are effective against multiple isolates of SARS-CoV-2. Berberine, a plant-derived alkaloid, inhibited SARS-CoV-2 at low micromolar concentrations and obatoclax, which was originally developed as an anti-apoptotic protein antagonist, was effective at sub-micromolar concentrations. Time-of-addition studies indicated that berberine acts on the late stage of the viral life cycle. In agreement, berberine mildly affected viral RNA synthesis, but it strongly reduced infectious viral titers, leading to an increase in the particle-to-pfu ratio. In contrast, obatoclax acted at the early stage of the infection, which is in line with its activity to neutralize the acidic environment in endosomes. We assessed infection of primary human nasal epithelial cells that were cultured on an air-liquid interface and found that SARS-CoV-2 infection induced and repressed expression of specific sets of cytokines and chemokines. Moreover, both obatoclax and berberine inhibited SARS-CoV-2 replication in these primary target cells. We propose berberine and obatoclax as potential antiviral drugs against SARS-CoV-2 that could be considered for further efficacy testing.

## 1. Introduction

Coronaviruses form a group of respiratory viruses in the order *Nidovirales* that possess a positive-sense RNA genome of approximately 30 kb [1]. Two coronaviruses of zoonotic origin caused significant outbreaks in the near past, severe acute respiratory syndrome coronavirus (SARS-CoV) [2,3] and Middle Eastern respiratory syndrome coronavirus (MERS-CoV) [4]. In late 2019, a new coronavirus that was closely related to SARS-CoV emerged in Wuhan, China, and rapidly spread across the globe. As of today, more than 100 million SARS-CoV-2 cases have been confirmed worldwide, but the total number may be up to 20 times higher due to asymptomatic and undetected cases [5,6] (WHO Coronavirus Disease Dashboard, https://covid19.who.int/, accessed on 07 February 2021).

SARS-CoV-2 infects epithelial cells in the nasal and oral cavities via binding of the spike protein to the ACE2 receptor [7]. The spike glycoprotein requires cleavage by the cell-surface protease TMPRSS2 in order to convert it into an active form, allowing for fusion at the plasma membrane [7,8,9,10]. Alternatively, in permissive cells lacking TMPRSS2, receptor binding is most likely followed by dynamin and clathrin-mediated endocytosis of the virus particle into endosomal compartments [11]. Acidification of these compartments leads to the activation of cathepsin-B/L proteases that cleave the spike protein, initiating membrane fusion and release of the encapsidated viral RNA into the cytoplasm [12,13,14]. Upon nucleocapsid disassembly, the viral positive-sense RNA genome is translated into two open reading frames that encode for several non-structural proteins (reviewed in [15,16,17]). These proteins constitute the machinery that replicates the viral RNA and transcribes subgenomic RNAs that code for viral structural proteins and several accessory proteins [16]. Newly formed viral genomic RNA is coated with nucleocapsid proteins, which interact with the structural proteins, resulting in budding into, and transit through, the ER-Golgi network and the release of mature viral particles through exocytosis [16,17]. The virus then spreads to the lower respiratory tract and infects alveolar type-II pneumocytes in the lungs, where a severe infection can lead to acute respiratory distress syndrome [18].

A year into the COVID-19 pandemic, there has been an unprecedented fast development of vaccines [19], as well as study of antiviral or adjunctive host-directed therapy. However, apart from dexamethasone [20], none have been proved effective. There is a clear need for improved antiviral therapy, as preventive measures, including vaccines, are unlikely to curb the epidemic alone. This could be applied either preventively, or after symptom development.

In this study, we assessed the plant-based alkaloid berberine and obatoclax, an anticancer drug that has proven safe in clinical trials, as possible antiviral candidates against SARS-CoV-2. Berberine (BBR) [21,22,23] and obatoclax (OLX) [24,25] have both broad-spectrum antiviral activity against a range of different viruses, including herpes simplex virus, influenza A virus, chikungunya virus, and Zika virus. We show that these compounds are effective against two different isolates of SARS-CoV-2 at low micromolar concentrations with promising selectivity indices. Antiviral activity was observed in Vero E6 cells as well as in physiologically relevant nasal epithelial cells that are cultured on an air-liquid interface. We propose these compounds for further assessment as antiviral agents against SARS-CoV-2.

## 2. Materials and Methods

### 2.1. Cells

African green monkey Vero E6 (ATCC CRL-1586) and Vero FM (ATCC CCL-81) kidney epithelial cells were grown in Dulbecco’s modified Eagle medium containing 4.5 g/L glucose and L-glutamine (Gibco, Thermo Fisher Scientific, Waltham, MA), which was supplemented with 10% fetal calf serum (FCS, Sigma Aldrich), 100 µg/mL streptomycin, and 100 U/mL penicillin (Gibco). The cells were maintained at 37 °C with 5% CO_2_.

Primary epithelial cells were derived from post-operative residual tissue from the posterior nasal septum of a 17-year-old male, which was obtained after informed consent, according to the principles of the declaration of Helsinki. The patient had a fully normal nasal epithelium, was SARS-CoV-2 negative, and had no history of allergy, (chronic) rhinosinusitis, or other mucosal disease. A single cell suspension was made by incubating the tissue in Hank’s Balanced Salt Solution (Gibco) with 1 mg/mL collagenase from clostridium histolyticum (Sigma-Aldrich, St. Louis, MO) and 0.02 mg/mL DNase 1 (Sigma-Aldrich) for 2 h at 37 °C. The cell suspension was filtered through a 70 µm cell strainer and the flow-through was collected. The cells were cultured and expanded in PneumaCult-Ex Basal plus Medium with supplements (Stemcell Technologies, Vancouver, Canada), 0.48 µg/mL hydrocortisone (Stemcell), and 100 µg/mL streptomycin and 100 U/mL penicillin (Lonza, Basel, Switzerland), according to the manufacturer’s protocol (Stemcell). 50,000 primary nasal epithelial cells were seeded on 0.4 μm pore polyester membrane inserts (Corning) and expanded for one week in PneumaCult-Ex Basal plus Medium at 37 °C and 5% CO_2_. When the cells had formed a tight epithelial layer, they were cultured on an air-liquid interface by discarding the apical medium and adding PneumaCult-ALI Basal Medium with supplements (Stemcell), 4 µg/mL heparin (Stemcell), 0.48 µg/mL hydrocortisone (Stemcell), and 100 µg/mL streptomycin and 100 U/mL penicillin (Lonza) to the basal compartment to induce differentiation. The cells were differentiated for four weeks on this air-liquid interface. Prior to experiments, the cells were cultured for three days in PneumaCult-ALI Basal Medium without heparin, hydrocortisone, and antibiotics [26].

### 2.2. Viruses

SARS-CoV-2 isolate BetaCoV/Munich/BavPat1/2020 (European Virus Archive no. 026V-03883) was kindly provided by Prof. C. Drosten (Charité-Universitätsmedizin, Berlin Institute of Virology, Berlin, Germany), and it was initially cultured in Vero E6 cells up to three passages in the laboratory of Prof. B. Haagmans (Viroscience Department, Erasmus Medical Center, Rotterdam, The Netherlands). To prepare virus stock, Vero FM cells were infected with passage 3 stock at an MOI of 0.01 in virus infection medium [DMEM (Gibco) containing 2% FCS (Sigma-Aldrich), 20 mM HEPES buffer (Gibco), 100 µg/mL streptomycin (Gibco), and 100 IU/mL penicillin (Gibco)]. At 48 h post infection (hpi), cell culture supernatant was harvested, centrifuged at 4700× *g* for 10 min. at 4 °C to remove cellular debris, filtered through a 0.2 µm syringe filter (Whatman, Maidstone, UK), and then stored as 100 µL aliquots at −80 °C.

Isolate SARS-CoV-2/human/NLD/Nijmegen1/2020 was isolated from an oro-nasopharyngeal swab of a 65-year old male COVID-19 patient that was hospitalized at Radboud University Medical Center in May 2020, collected as part of an ongoing study on COVID-19 infectiousness and viral kinetics. The Committee on Research Involving Human Subjects Arnhem Nijmegen (CMO NL2020-6517) approved the study and it conducted according to the principles of the Declaration of Helsinki (last updated 2013) and in accordance with the Medical Research Involving Human Subjects Act (Dutch: WMO). Verbal consent was provided at inclusion and separate verbal consent was obtained for the use of isolated virus in additional experiments.

The nasal swab was initially stored in virus transport medium [Hank’s Balanced Salt Solution (Gibco) containing 2% FCS (Sigma-Aldrich), 100 µg/mL gentamycin (Gibco), and 0.5 µg/mL amphotericin-B (Gibco)]. Vero FM cells that were seeded in 24-well plates were infected in duplicate with 100 µL two-fold serial dilutions of patient material in a total volume of 200 µL using virus isolation medium [DMEM (Gibco) containing, 20 mM HEPES buffer (Gibco), 100 µg/mL streptomycin (Gibco), 100 IU/mL penicillin (Gibco), 1% amphotericin B (Gibco)]. After 1 h adsorption, viral inoculum was discarded, cells were washed with PBS, and 500 µL virus isolation medium containing 2% FCS was added. 100 µL of supernatant was collected at two, four, and six days post-infection, subjected to RNA isolation and RT-qPCR to detect SARS-CoV-2 RNA. The rest of the supernatants were stored at −80 °C. The supernatant from which a positive signal was obtained in RT-qPCR was titrated by conventional plaque assay and it was cultured in Vero FM cells at an MOI of 0.01 to obtain passage 1 working stocks. The near full-length viral sequence of the isolate was deduced by amplicon-based next-generation sequencing [27], supplemented with Sanger sequencing to fill few remaining gaps in the obtained sequence. The sequence has been deposited in GenBank under accession number MW577029 and it was assigned lineage B.1, using the web application of the Pangolin COVID-19 Lineage Assigner (https://pangolin.cog-uk.io/, accessed on 07 February 2021) [28].

### 2.3. Virus Titration

Vero E6 cells were seeded onto 12-well plates at a density of 5 × 10^5^ cells/well. At 24 h post-seeding, the cells were washed twice with PBS and then infected with 200 µL 10-fold serial dilutions of the virus. After 1 h adsorption, the inoculum was discarded, cells were washed with PBS, and overlay medium containing Minimum essential medium (MEM, Gibco), 2% FCS (Sigma-Aldrich), 20 mM HEPES buffer (Gibco), 0.75% carboxymethyl cellulose (Sigma-Aldrich), 100 µg/mL streptomycin, and 100 IU/mL penicillin (Gibco) was added onto the cells. At 48 hpi, the medium was discarded, cells were washed with PBS, and then stained with 0.25% crystal violet solution containing 4% formaldehyde for 30 min. The plates were then washed with double-distilled water, dried and plaques were counted.

### 2.4. Infection of Primary Nasal Epithelial Cells

Differentiated primary nasal epithelial cells seeded onto 0.4 µm pore polyester membrane inserts (Corning, Corning, NY, USA) were inoculated with SARS-CoV-2 BavPat1 isolate at an MOI of 10 at both the apical and basolateral surfaces. At 2 hpi, the inoculum was discarded, cells were washed with PBS, and fresh PneumaCult-ALI Basal Medium (Stemcell) without heparin, hydrocortisone, and antibiotics was added to the basolateral compartment. At the desired time points, 200 µL of pre-warmed medium was added to the apical surface of the cells and then incubated for 10 min. at 37 °C. Apical and basolateral supernatants were collected for titration or RNA isolation. Intracellular RNA was isolated using RNA-Solv reagent (Omega Bio-Tek, Norcross, GA) according to the manufacturer’s protocols, while using glycogen during precipitation.

### 2.5. Antiviral Assays

BBR (Sigma-Aldrich) and OLX (Selleck Chemicals, Houston, TX) were dissolved in DMSO at a stock concentration of 10 mM. Vero E6 cells were seeded onto 24-well plates at a density of 1.5 × 10^5^ cells/well. 24 h post-seeding, the cells were washed twice with PBS and then infected with the SARS-CoV-2 BavPat1 or Nijmegen1 isolate at an MOI 0.01 in the presence of eight concentrations of a two-fold dilution series of BBR or OLX. As a negative control, 0.1% DMSO was used, corresponding to the DMSO concentration in cells that were treated with 10 µM of compound. At 1 hpi, virus inoculum was discarded, cells were washed with PBS and replaced with infection medium containing the same concentrations of the inhibitors. At 24 hpi, the cell culture supernatants were collected and stored at −80 °C for plaque titration. RNA was isolated from cells or 100 µL cell culture supernatant using RNA-Solv reagent (Omega Bio-Tek) and precipitated in the presence of glycogen.

Differentiated primary nasal epithelial cells, which were seeded onto 0.4 µm pore polyester membrane inserts (Corning), were infected with SARS-CoV-2 BavPat1 isolate at an MOI of 10 at both the apical and basolateral surfaces in the presence of a two-fold dilution series of BBR or OLX. At 2 hpi, the inoculum was discarded, cells were washed with PBS, and fresh PneumaCult-ALI Basal Medium without heparin, hydrocortisone, and antibiotics, containing the same concentrations of BBR or OLX was added to the basolateral compartment. SARS-CoV-2 infection in the presence of 0.1% DMSO was used as a negative control. At 72 hpi, 200 µL of pre-warmed medium was added to the apical surface of the cells and then incubated for 10 min. at 37 °C and 200 µL was collected for RNA isolation. The effective concentration of compound that reduced viral levels by 50% (EC_50_) was estimated by four parameter logistic regression, using Graphpad Prism (version 5.0). The selectivity index (SI) was defined as the CC_50_/EC_50_ ratio.

### 2.6. Cell Viability Assay

Vero E6 cells that were seeded in 96-well plates at a density of 3 × 10^4^ cells/well were treated with two-fold dilution series of BBR and OLX in the absence of SARS-CoV-2 infection. Cells treated with 0.1% DMSO were used as a negative control. At 24 h post-treatment, the cell viability was assessed using the Cell Titer Glo 2.0 kit (Promega, Madison, WI, USA) according to the manufacturer’s instructions. Luminescence was detected on the Victor Multilabel Plate Reader (Perkin Elmer, Waltham, MA, USA). The 50% cytotoxicity concentration (CC_50_) value was estimated by four parameter logistic regression of the data, using Graphpad Prism (version 5.0).

### 2.7. Time-of-Addition Assay

Vero E6 cells were seeded in 24-well plates at a density of 1.5 × 10^4^ cells/well. At 24 h post-seeding, the cells were washed twice with PBS and then infected with SARS-CoV-2 BavPat1 isolate at an MOI of 1. At indicated time points during the course of the experiment, 20 µM BBR, 0.25 µM OLX, or 0.1% DMSO was added. Cell culture supernatants were collected at 10 hpi and infectious viral titers were analyzed and estimated by plaque assay.

### 2.8. RT-qPCR

TaqMan Reverse Transcription reagent and random hexamers (Applied Biosystems, Foster City, CA, USA) were used for cDNA synthesis. Semi-quantitative real-time PCR was performed using GoTaq qPCR Master Mix (Promega, Madison, WI, USA) while using primers targeting the SARS-CoV-2 E protein gene (forward primer, 5′-ACAGGTACGTTAATAGTTAATAGCGT-3′; reverse primer, 5′-ACAGGTACGTTAATAGTTAATAGCGT-3′). A standard curve of a plasmid containing the E gene qPCR amplicon was used to convert Ct values to relative genome copy numbers. Human and African green monkey (Chlorocebus sabaeus) β-actin were used as housekeeping genes for normalization (Human β-actin, forward primer 5′-CCTTCCTGGGCATGGAGTCCTG-3′, and reverse primer 5′-GGAGCAATGATCTTGATCTTC-3′; African green monkey β-actin, forward primer 5′- ATTGGCAATGAGCGGTTCC-3′, and reverse primer 5′-CTGTCAGCAATGCCAGGGTA-3′).

### 2.9. Immunofluorescence Staining

Primary nasal epithelial cells were mock-infected with PneumaCult-ALI Basal Medium for 2 h or infected with SARS-CoV-2 BavPat1 isolate at an MOI of 10. Vero E6 cells were infected with SARS-CoV-2 BavPat1 isolate at an MOI of 0.01 or mock-infected for 1 h at 37 °C, after which the cells were washed with PBS. The primary nasal epithelial cells were fixed with 4% formaldehyde at 0, 24, 48, 72, and 96 hpi. Vero E6 cells were fixed at 24 hpi. Cells were permeabilized with 0.1% Triton X-100 for 5 min., blocked with 2% Normal Serum Block (BioLegend, San Diego, CA, USA), 1% BSA, and 0.0005% Triton X-100 in PBS for 30 min. The primary nasal epithelial cells and Vero E6 cells were stained for 1 h with 0.01 mg/mL rabbit anti-SARS-CoV-2 Spike S1 subunit (clone#007, Sino Biologicals, Bejing, China) and 0.03 mg/mL mouse anti-Tubulin IV (cloneONS.1A6, Sigma) or mouse anti-Muc5AC (clone 45M1, Invitrogen, Carlsbad, CA, USA). Subsequently, the cells were stained for 1 h with 0.01 mg/mL goat anti-mouse Dylight 488 (Biolegend), 0.01 mg/mL donkey anti-rabbit AF555 (Biolegend), and a 1:2000 dilution of phalloidin-iFluor 647 reagent (Abcam). For Figure S1, Vero E6 cells (Figure S1) were stained for 1 h with anti-SARS-CoV Spike S1 subunit human IgG1 (BEI Resources) and mouse anti-dsRNA J2 monoclonal antibody (Scicons, Szirák, Hungary). Goat anti-mouse Alexa Fluor 568 (Invitrogen) and goat anti-human Alexa Fluor 488 (Invitrogen) were used at 1:500 dilutions for secondary staining. The polyester membrane containing the nasal epithelial cells was cut out of the insert with a scalpel and then placed on a microscopy slide, embedded in ProLong Diamond Antifade Mountant with DAPI (Invitrogen), and covered with a coverslip. Vero E6 that had been grown on coverslips was embedded in the same way. Fluorescent images were made using a Leica Dmi8 microscope with 20× and 100× objectives and Leica CFC7000 GT camera using LAS X 3.4.2 software. Confocal images for Vero E6 cells in Figure S1B were captured using a Zeiss LSM9000 microscope with a 63× oil objective and then processed using FIJI software.

### 2.10. Cytokine and Chemokine Analysis

Primary nasal epithelial cells that were cultured on an air-liquid interface were infected with SARS-CoV-2 (BavPat1 isolate) at an MOI of 10 or mock infected. Samples were taken from the basolateral medium over time and the concentration of chemokines and cytokines was determined using the LEGENDplex Human Anti-Virus Response Panel (13-plex) and the LEGENDplex Human Proinflammatory Chemokine Panel (13-plex) on a FACSCanto II flow cytometer (BD). The samples were run in duplicate and measured at two different dilutions.

## 3. Results

### 3.1. Berberine and Obatoclax Inhibit SARS-CoV-2 Replication

BBR and OLX have shown antiviral activity against viruses from multiple families [21,22,23,24,25]. Therefore, we tested these compounds against SARS-CoV-2 in Vero E6 cells. A virus growth curve was performed to determine the optimal time point for the assay. At a low multiplicity of infection (MOI) of 0.01, SARS-CoV-2 titers peaked at 24 h post-infection (hpi) (Figure S1A). The robust infection of Vero E6 cells was confirmed through staining infected cells for the presence of dsRNA replication intermediates and the SARS-CoV-2 spike protein. dsRNA staining was observed in the perinuclear area, likely corresponding to the ER-Golgi network containing SARS-CoV-2 replication complexes (Figure S1B). Spike protein expression was also detected in the perinuclear area, where they are synthesized, but also on the outer periphery of the cells, probably from viruses exiting the infected cells.

**Figure 1:**
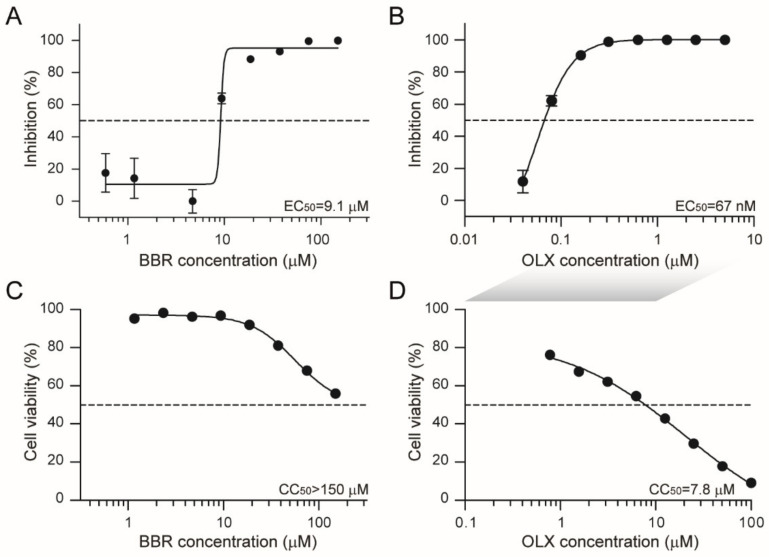
Berberine and obatoclax are effective antiviral compounds against Severe acute respiratory syndrome coronavirus 2 (SARS-CoV-2). Vero E6 cells were infected with SARS-CoV-2 (BavPat1 isolate) at an MOI of 0.01 for 24 h in the presence of the indicated concentrations of (**A**) berberine (BBR) or (**B**) obatoclax (OLX) in a two-fold dilution series or 0.1% DMSO as a control. Infectious viral titers from duplicate cell culture supernatants were assessed by plaque assay and plotted as percentage inhibition compared to the DMSO control. Error bars indicate SD. Dashed lines indicate 50% inhibition. A representative of two independent experiments is shown. Vero E6 cells were treated with the indicated concentrations of (**C**) berberine or (**D**) obatoclax or 0.1% DMSO. 24 h post-treatment, ATP content in cells from triplicate wells was measured as indication for cell viability, plotted as percentage compared to the DMSO control. Error bars indicate SD. A representative of two independent experiments is shown. The shaded grey box indicates that different OLX concentrations were used for antiviral (B) and viability assays (D).

Next, a dose response assay was carried out under these conditions with a series of concentrations of BBR and OLX, using 0.1% DMSO as a negative control. Plaque assay titrations of the viral supernatants showed that both BBR and OLX are effective against SARS-CoV-2 with 50% effective concentration (EC_50_) values at low micromolar and nanomolar concentrations (EC_50 BBR_ = 9.1 µM and EC_50 OLX_ = 67 nM, respectively; Figure 1A,B). The cytotoxicity of these compounds was tested under the same conditions, but in the absence of SARS-CoV-2 infection. BBR was only toxic at the highest concentrations tested (50% cellular cytotoxicity, CC_50_ > 150 µM), resulting in a selectivity index of >16 (Figure 1C), whereas the CC_50_ value for OLX was 7.8 µM (Figure 1D), corresponding to a high selectivity index of 116, due to its low EC_50_ value. Overall, these results indicate that BBR and OLX show potent antiviral activity against SARS-CoV-2 with good selectivity indices.

### 3.2. Berberine and Obatoclax Are Effective Against a SARS-Cov-2 Isolate from a Different Geographic Region

**Figure 2:**
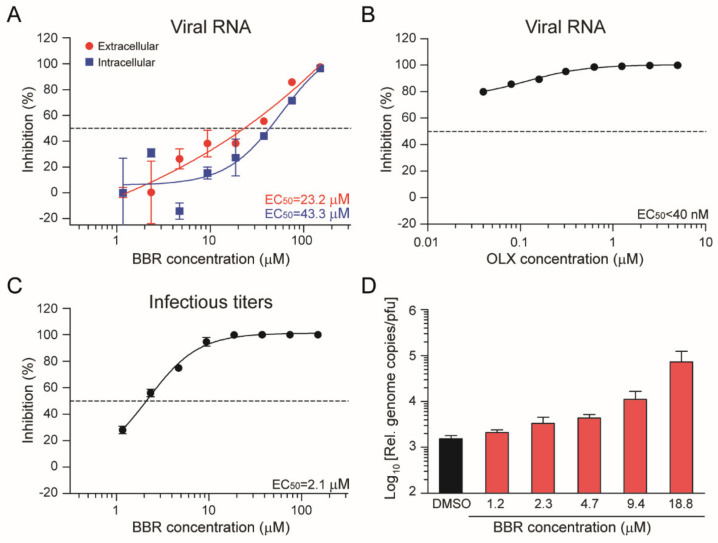
Berberine and obatoclax are effective against SARS-CoV-2 Nijmegen1 isolate. Vero E6 cells were infected with the SARS-CoV-2 Nijmegen1 isolate at an MOI of 0.01 for 24 h in the presence of the indicated concentrations of (**A**–**C**) berberine (BBR) or (**B**) obatoclax (OLX) in a two-fold dilution series or 0.1% DMSO as a control. (**A**,**B**) Viral RNA from duplicate cell culture supernatants (red line in panel A) was quantified by qRT-PCR and plotted as percentage inhibition compared to the DMSO control. For BBR-treated cells, both extracellular and Intracellular viral RNA was analyzed (red and blue lines, respectively). Intracellular viral RNA levels were normalized to the human β-actin housekeeping gene. (**C**) Infectious viral titers from duplicate cell culture supernatants were quantified by plaque assay and plotted as percentage inhibition compared to the DMSO control. Error bars indicate SD. Dashed line indicates 50% inhibition. (**D**) The ratio of relative genome copies to infectious viral particles in cell culture supernatants for the indicated, non-toxic BBR concentrations and DMSO control.

Our initial antiviral assays were performed using a SARS-CoV-2 isolate from Bavaria, Germany (BavPat1). We isolated a SARS-CoV-2 isolate from a hospitalized COVID-19 patient at Radboud University Medical Center, the Netherlands to assess whether BBR and OLX are also effective against another SARS-CoV-2 isolate. This isolate, SARS-CoV-2/human/NLD/Nijmegen1/2020 (hereafter called Nijmegen1), had a relatively small plaque phenotype (Figure S2), in agreement with the short passage history in Vero cells [29,30]. We used the Nijmegen1 isolate to perform dose response assays with BBR and OLX, using viral RNA in the cell culture supernatants as a readout (Figure 2). OLX showed stronger antiviral activity for the Nijmegen1 isolate (EC_50_ = < 0.04 µM) (Figure 2B) as compared to the BavPat1 isolate. In contrast, BBR showed relatively weak antiviral activity against the Nijmegen1 isolate (EC_50_ = 23.2 µM for viral RNA in the supernatant and EC_50_ = 43.3 µM for intracellular viral RNA; Figure 2A) when compared to the initial experiments in which infectious titers were used as a readout. Therefore, we used conventional plaque assay to assess the antiviral activity of BBR against the SARS-CoV-2 Nijmegen1 isolate. We observed strong antiviral activity (EC_50_ = 2.1 µM) (Figure 2C), which was slightly lower than for the BavPat1 isolate.

These results suggest that BBR does not inhibit viral RNA replication per se, but that it affects the production of infectious virus. To quantify this, we calculated the relative genome copy to infectious virus particle ratio for the different concentrations of berberine. Indeed, when compared to the DMSO control, this ratio increased upon BBR treatment in a dose-dependent manner (Figure 2D). Altogether, these results confirmed that both BBR and OLX are effective against a second, early passage isolate of SARS-CoV-2. Moreover, we conclude that BBR does not affect viral RNA replication or the secretion of viral particles, but that it strongly affects the infectivity of the virus particles produced.

### 3.3. Berberine and Obatoclax Act at Different Stages of The SARS-Cov-2 Life Cycle

**Figure 3:**
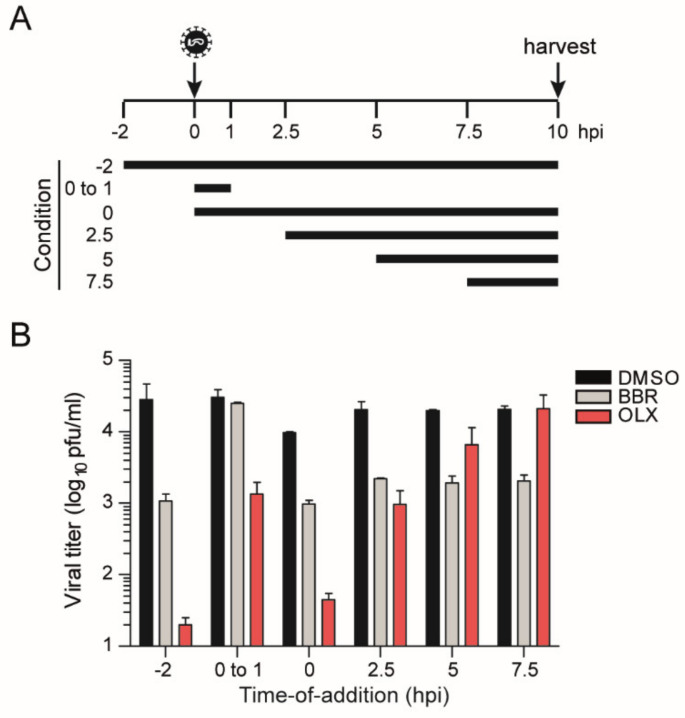
Time-of-addition assay. Vero E6 cells were infected with SARS-CoV-2 (BavPat1 isolate) at an MOI of 1. (**A**) Schematic layout of the assay. 20 µM berberine (BBR), 0.25 µM obatoclax (OLX), or 0.1% DMSO as a control was added to the infected cells at the indicated time points. (**B**) Plaque assay titers from cell culture supernatants that were collected at 10 hpi. Bars and error bars represent means and SD of n = 2 replicates.

We performed a time-of-addition assay to decipher a putative mode of action for BBR and OLX. Vero E6 cells were infected at an MOI of 1, adding 20 µM BBR or 0.25 µM OLX at different time points during the course of the experiment (Figure 3A). The cell culture supernatants were harvested at 10 hpi and infectious viral titers were measured by plaque assay. OLX showed a very potent inhibition of three logs at the early stages of the viral life cycle. The levels of inhibition gradually decrease as OLX is added at later time points of the experiment (Figure 3B), which suggests that OLX most likely affects the early stages of the SARS-CoV-2 infection cycle. Indeed, when OLX was added during the 1 h of virus adsorption and subsequently discontinued, it reduced the viral titers by 1.5 logs, indicating that the compound inhibits viral entry.

For BBR, an inhibition of almost 1.5 logs was seen when the compound was added to the cells 2 h prior to inoculation and maintained throughout the experiment. However, no inhibition was observed when BBR is only added during the 1 h of virus adsorption. The potent inhibition of 1 log was observed when BBR was added during inoculation and continuously maintained thereafter. Strikingly, BBR continues to be equally effective at reducing viral titers, even when added at 7.5 hpi, indicating that it acts late in the viral infection cycle (Figure 3B), in line with our conclusion that BBR affects the production of infectious virus particles (Figure 2).

### 3.4. SARS-CoV-2 Infection of Primary Nasal Epithelial Cells

Even though SARS-CoV-2 efficiently replicates in Vero E6 cells, these cells are derived from the African green monkey kidney, have a defective interferon pathway [31], and do not express TMPRSS2 [32], and, thus, they are not representative of natural target cells. Therefore, it is essential to validate candidate antiviral compounds in human host cells [14]. Therefore, we cultured and differentiated human nasal epithelial cells, which were residual after surgery, on an air-liquid interface. This approach to mimic the natural environment of the infected host has previously been used for studying circulating seasonal human coronaviruses, such as HCoV-229E, HCoV-OC43, HCoV-NL63, and HCoV-HKU1, as well as the zoonotic SARS and MERS coronaviruses [33].

**Figure 4:**
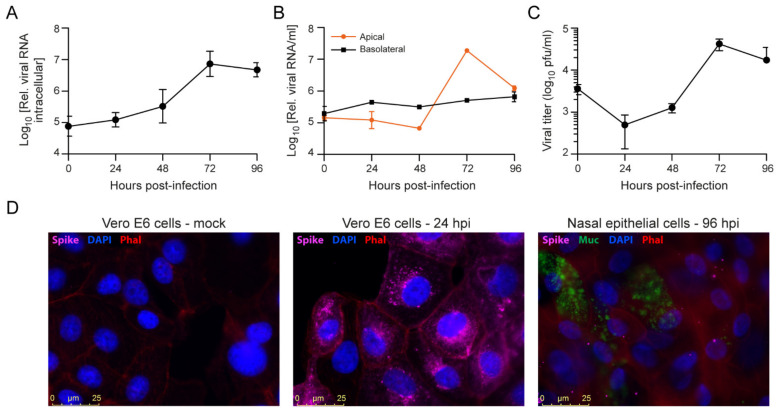
SARS-CoV-2 infection of primary nasal epithelial cells. Primary nasal epithelial cells, cultured on an air-liquid interface, were infected with SARS-CoV-2 (BavPat1 isolate) at an MOI of 10. At the indicated time points, cells were harvested and viral RNA from (**A**) cells or (**B**) apical and basolateral compartment supernatants was quantified by RT-qPCR. Intracellular viral RNA levels were normalized to the human β-actin housekeeping gene. Error bars indicate SD (n = 2). (**C**) Infectious viral titers from apical supernatants corresponding to the indicated time points were quantified by plaque assay. Error bars indicate SD (n = 2). (**D**) Immunofluorescence staining of mock (left panel), SARS-CoV-2 infected Vero E6 cells fixed at 24 hpi (MOI = 0.01; middle panel) or SARS-CoV-2 infected primary nasal epithelial cells fixed at 96 hpi (MOI = 10; right panel). The presence of SARS-CoV-2 Spike protein subunit S1 (pink) was assessed in cells stained with DAPI (nucleus, blue) and phalloidin (F-actin, red). Nasal epithelial cells were additionally stained with anti-Muc5AC antibodies (Muc, goblet cells, green). Bar represents 25 µm.

Virus replication was assessed to determine the optimal time point for antiviral assays. We analyzed intracellular RNA, extracellular RNA from apical and basal supernatants, infectious viral titers, and viral protein expression at different time points post-infection. Peak intracellular viral RNA levels were detected at 72 hpi (Figure 4A). No time-dependent increase in viral RNA levels was observed in basolateral supernatants, which suggested the inefficient release of virus into this compartment. In contrast, viral RNA signals from the apical surface supernatants were at their highest at 72 hpi, corresponding to the kinetics of intracellular RNA (Figure 4B). The preferential release from the apical membrane in polarized epithelial cells has been observed before for multiple viruses, including SARS-CoV [34,35,36]. Infectious viral titers from the apical supernatants also showed active viral replication, with peak titers at 72 hpi (Figure 4C) in line with the viral RNA kinetics (Figure 4A,B).

The nasal epithelium contains both ciliated cells and mucus producing goblet cells after four weeks of differentiation on the air-liquid interface (Figure S3). We analyzed SARS-CoV-2 spike protein expression by immunofluorescence microscopy to assess infection of these cell types. To validate the anti-Spike protein antibody used, we first analyzed SARS-CoV-2 infected Vero E6 cells and observed abundant S protein expression in the perinuclear area as well as the periphery in order (Figure 4D). In SARS-CoV-2 infected nasal epithelial cells, SARS-CoV-2 S protein was detected from 48–72 hpi onwards (Figure S3). In these cells, the S protein was mostly located at the outer cell periphery and, as expected, at much lower levels than in Vero E6 cells. At 0 hpi, some S protein staining is also visible, which likely corresponds to viral particles that have not internalized and remain attached to the cell surface (Figure S3). Additionally, the epithelial cells were stained for the differentiation markers tubulin IV and Muc5Ac to identify ciliated cells and goblet cells, respectively. It did not appear that viral infection was more abundant in either of these cell types (Figure 4D, Figure S3).

### 3.5. Cytokine and Chemokine Responses Are Selectively Enhanced or Inhibited during SARS-Cov-2 Infection of Primary Nasal Epithelial Cells

**Figure 5:**
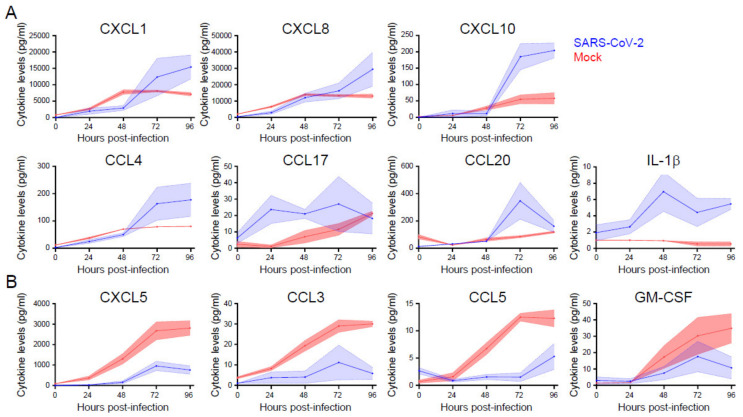
The induction and repression of specific cytokine and chemokines during SARS-CoV-2 infection of primary nasal epithelial cells. Primary nasal epithelial cells cultured on an air-liquid interface were infected with SARS-CoV-2 (BavPat1 isolate) at an MOI of 10 or mock infected. At the indicated time points, medium from the basolateral compartment was harvested and the concentration of the indicated cytokines and chemokines was analyzed by a bead-based immunoassay. Cytokines and chemokines with increased (**A**) and decreased (**B**) expression are shown. Means and SEM (shading) of n = 2 replicates are shown.

We measured cytokine and chemokine concentrations in the cell culture medium of primary nasal epithelial cells that were infected with SARS-CoV-2 from 0 to 96 hpi to assess the epithelial (immune) response to SARS-CoV-2 infection. Cytokines and chemokines that are associated with granulocyte (CXCL1 and CXCL8), monocyte (CXCL10, CCL4, and CCL20), NK cell (CXCL10 and CCL4), and T cell (CCL20, CCL17, and CXCL10) chemotaxis and activation increased over time when compared to the mock infected cells (Figure 5A). CXCL1, CXCL8, CXCL10, CCL4, and CCL20 showed a more rapid production rate between 48 and 96 hpi, which is in line with the viral replication kinetics that we observed (Figure 4), suggesting that viral replication triggers cytokine and chemokine production in these cells. Strikingly, the cytokines that are typically associated with viral infection and acute phase reactions (IL-1β, TNF-α2, IL-6, IFN-λ1, and IFN-λ2,3) did not show increased production when compared to mock infected cells, with the exception of IL-1β (Figure 5A and Figure S4). Furthermore, within the first 48 h of infection, the production of CXCL5, CCL3, CCL5, and, to a lesser extent, GM-CSF, was reduced (Figure 5B). This could be indicative of immune evasive activity of SARS-CoV-2.

These data suggest that the epithelium selectively stimulates cellular immune responses in response to SARS-CoV-2 infection, while the virus may inhibit the secretion of specific cytokines. Both of the processes appear to be selective, as for various other cytokines, no difference in production could be observed between infected cells and mock infected cells (Figure S4). Other cytokines that we tested, such as IFN-α, IFN-β, IL-10, and IL-12p70, were not produced by the epithelium at all (data not shown). Together, these data indicate a specific and dynamic interaction between airway epithelial cells and SARS-CoV-2.

### 3.6. Berberine and Obatoclax Are Effective Against SARS-Cov-2 in Nasal Epithelial Cells

**Figure 6:**
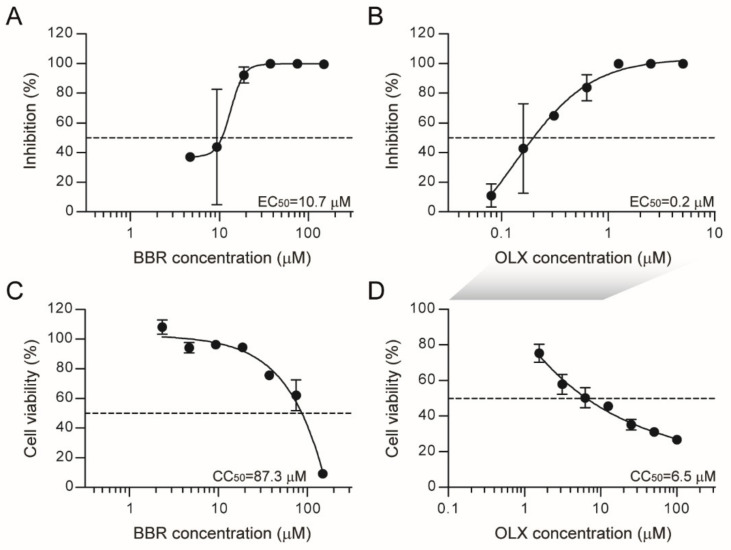
Berberine and obatoclax inhibit SARS-CoV-2 replication in primary nasal epithelial cells. Primary nasal epithelial cells cultured on an air-liquid interface were infected with SARS-CoV-2 (BavPat1 isolate) at an MOI of 10 in the presence of the indicated concentrations of (**A**) berberine (BBR) or (**B**) obatoclax (OLX) in a two-fold dilution series or 0.1% DMSO as a control. Viral RNA from duplicate apical surface supernatants at 72 hpi was quantified by qRT-PCR and plotted as percentage inhibition compared to the DMSO control. Error bars indicate SD (n = 2). Dashed line indicates 50% inhibition. Primary nasal epithelial cells cultured on an air-liquid interface were treated with the indicated concentrations of (**C**) BBR or (**D**) OLX or 0.1% DMSO. At 72 h, the ATP content in cells from duplicate samples was measured as a measure of cell viability, plotted as a percentage of the DMSO control. Error bars indicate SD. Dashed lines indicate 50% viability. A representative of two independent experiments is shown. The shaded grey box indicates that different OLX concentrations were used for antiviral (B) and viability assays (D).

Having established primary nasal epithelial cells as a relevant cellular model for SARS-CoV-2 infection, we assessed the antiviral activity of BBR and OBX in these cells. The nasal epithelial cells were infected with SARS-CoV-2 in the presence of increasing concentrations of BBR or OLX, along with DMSO as a negative control, and viral RNA levels were assessed at 72 hpi. BBR was effective in inhibiting SARS-CoV-2 RNA levels in the supernatant of this nasal epithelial cell model with an EC_50_ value of 10.7 µM (Figure 6A), similar to that seen in Vero E6 cells. Likewise, OLX was effective in the sub-micromolar range with an EC_50_ value of 0.2 µM. Cytotoxicity assays were done with BBR and OLX over a 72 h time period. There was a slight increase in toxicity in these cells as compared to the shorter 24 h exposure period in Vero E6 cells (Figure 6C,D), which resulted in lower selectivity indices for both compounds (SI = 8.1 and 32.7 for BBR and OLX, respectively) than in Vero E6 cells. Altogether, these results indicate that BBR and OLX effectively inhibit SARS-CoV-2 replication in a physiologically relevant human nasal epithelial cell model.

## 4. Discussion

The COVID-19 pandemic has rapidly spread across the world, causing large-scale morbidity and mortality [5] (WHO Coronavirus Disease Dashboard, (https://covid19.who.int/, accessed on 07 February 2021) and huge economic losses [37]. Effective treatment for patients who fall ill despite vaccination or other preventive measures is urgently needed. In this study, we propose repurposing BBR and OLX, two compounds with good safety profiles in clinical trials, as potential therapeutic options against SARS-CoV-2.

BBR is an isoquinoline alkaloid that is derived from the Chinese herb *Coptis chinensis* and plants of the *Berberis* genus [38]. Its wide-ranging biological properties identified in pre-clinical studies include anti-inflammatory, anti-arrhythmic, antimicrobial [39], and cholesterol-lowering [40] activity. In a large phase IV study randomizing 612 patients, a treatment regimen containing 1000 mg BBR daily was not more effective in treating *Helicobacter pylori* than a comparator treatment, but it was well tolerated apart from a bitter taste (20%) and nausea (12%) as the most outspoken side effects [41]. Previous studies also identified a favorable safety profile, with predominantly mild gastrointestinal side effects [42]. BBR has not found a place in clinical practice, but five placebo-controlled clinical trials are currently recruiting patients to study the effect of BBR against a range of conditions, including colorectal adenomas, schizophrenia, and diabetes (ClinicalTrials.gov (accessed on 21 January 2021) Identifiers NCT03281096, NCT03333265, NCT03378934, NCT02808351, NCT02983188, NCT03976336, NCT03198572, and NCT02737943).

BBR has broad spectrum antiviral activity in vitro against viruses from several different families, including influenza A virus, enterovirus, chikungunya virus, hepatitis B and C viruses, HIV, respiratory syncytial virus, human cytomegalovirus, herpes simplex virus, and human papilloma virus (reviewed in [43]). Here, we show that BBR is effective against SARS-CoV-2 at low micromolar concentrations in Vero E6 cells. While this manuscript was in preparation, our results were confirmed by another study showing the antiviral activity of BBR against SARS-CoV-2 also in Vero E6 cells at a similar EC_50_ value (10.6 µM) [44]. Our study adds the validation of this finding in physiologically relevant primary nasal epithelial cells, where BBR is equally effective against SARS-CoV-2. Furthermore, we show that BBR acts late in the viral life cycle and that it likely induces the formation of non-infectious virus particles. Congruently, BBR also inhibited the replication of the alphaviruses Semliki Forest virus and chikungunya virus at a late stage [45], likely by interfering with capsid protein-viral RNA interactions, leading to nucleocapsid assembly or disassembly defects and less infectious viral particles [46]. It remains to be confirmed as to whether BBR has similar effects on assembly of SARS-CoV-2 virions.

In addition to inducing possible assembly affects, BBR may affect cellular pathways to reduce SARS-CoV-2 replication. BBR targets several cellular signaling pathways, including major MAP-kinase pathways (ERK, p38 MAPK, and JNK), as well as the NF-κB and AMPK/m-TOR signaling pathways [47,48]. Many viruses upregulate these pathways to maximally utilize cellular resources for their replication [49]. The modulation of these pathways by BBR has been implicated in its antiviral activity against several different viruses, including chikungunya virus [23], enterovirus 71 [22], and herpes simplex virus [21]. A recent proteomic analysis of SARS-CoV-2 interaction networks revealed that the p38 MAP kinase pathway was upregulated to counteract the host immune system and that inhibitors targeting this pathway had antiviral activity against SARS-CoV-2 [50]. The modulation of cellular pathways that are needed for productive virus infection could be a second mechanism, through which BBR inhibits SARS-CoV-2 replication.

OLX was originally developed as an inhibitor of Mcl-1, a member of the Bcl-2 family of anti-apoptotic proteins [51]. OLX has been through phase I and II trials for a range of malignancies. Convincing antitumor effects have, thus far, not been demonstrated, but obatoclax proved to be safe in doses of 20–40 mg/m^2^. The most frequently reported adverse events are dose-related grade 1 or 2 neurological symptoms shortly after infusion [52,53,54,55,56,57].

OLX has broad-spectrum antiviral activity, employing a mechanism independent of its pro-apoptotic activity. OLX acts as a weak base and it rapidly neutralizes the acidic environment of endosomes and endolysosomes [24]. The acidification of endosomes is required for entry by enveloped viruses of several different families, including SARS-CoV-2 in TMPRSS2-negative target cells (such as Vero cells), where endosomal cathepsin B/L proteases are activated by a drop in endosomal pH [7,13,58]. We found that OLX inhibited SARS-CoV-2 replication at sub-micromolar concentrations in Vero E6 cells (EC_50_ = 0.06 µM) with a very high selectivity index. This confirms the results of another study, in which OLX was reported to inhibit SARS-CoV-2-induced cytotoxicity in Vero E6 cells [59]. Our time-of-addition assays confirmed the early effect of OLX, in alignment with the expected mechanism of virus entry inhibition. Moreover, we observed antiviral activity in nasal epithelial cells, albeit with a three-fold higher EC_50_ value than in Vero E6 cells. This is probably due to SARS-CoV-2 entering airway epithelial cells at the plasma membrane, which depends on the cleavage of the viral spike protein by the TMPRSS2 protease, which is pH independent [7,58]. However, unlike chloroquine, which also increases the endosomal pH but does not inhibit SARS-CoV-2 replication in lung epithelial cells [14], we observed an antiviral effect with OLX. This observation suggests that OLX may affect other steps of the viral life cycle or that SARS-CoV-2 may, at least partly, also enter through the endosomal pathway in our experimental system. In clinical studies where OLX was intravenously administered over 3 h, plasma level concentrations of ~0.4 µM were obtained [52,53], suggesting that it would be possible to achieve therapeutic dose levels to treat COVID-19.

In order to identify candidate antiviral compounds, it is important to assess antiviral activity in physiologically relevant in vitro models, as drug sensitivities may be cell-type specific [14,60]. Therefore, we characterized SARS-CoV-2 infection in primary nasal epithelial cells, which are one of the first cells the virus encounters during infection [61]. Expectedly, in these cells with an active innate immune system, SARS-CoV-2 showed lower replication kinetics and reached lower titers than in Vero E6 cells. Still, a robust time-dependent increase in SARS-CoV-2 replication was seen using multiple assessment parameters—viral RNA levels, titers, and viral protein expression. Viral titers that we obtained correspond well to those previously observed in human tracheobronchial epithelial cells grown on an air-liquid interface [62]. In undifferentiated human airway epithelium, reconstituted from human primary nasal cells from a pool of 14 donors, higher SARS-CoV-2 titers were observed [63]. In contrast, we used differentiated cells derived from post-operative tissue of a single donor, which may contribute to the observed differences.

SARS-CoV-2 infection of primary nasal epithelial cells induced the epithelial release of CXCL1, CXCL8, CXCL10, CCL4, CCL17, CCL20, and IL1β. In contrast, we did not observe an increase in TNFα, IL-6, IFN-β, IFN-λ1, and IFN-λ2,3, cytokines that are typically involved in antiviral reactions and acute phase induction [64,65]. These observations might suggest that epithelial cells recruit leucocytes to the site of infection, rather than initiating an acute phase reaction themselves. Interestingly, viral infection also leads to reduced production of CXCL5, CCL5, CCL3, and GM-CSF, which are mainly involved in the attraction and activation of granulocytes and monocytes [66], suggesting that SARS-CoV-2 actively inhibits the recruitment of leukocytes early in infection. In contrast to our findings, Pizzorno et al. observed a minor increase in IL-1β and TNFα mRNA levels, and a larger increase in IFN-λ1 and IFN-λ2,3,4 mRNA levels in nasal epithelial cells that were infected with SARS-CoV-2 [63]. This discrepancy could be due to several differences in the experimental setup, including the readouts (intracellular RNA versus protein in the supernatant) and the source, anatomical location, and differentiation state of the cells [63].

In conclusion, our study puts forth two small molecule compounds, BBR and OLX, as repurposed antiviral molecules against SARS-CoV-2, in doses lower than that previously shown to be safe in human clinical trials. While OLX is effective at early steps of the viral life cycle, likely interfering with entry processes, BBR acts on the later stages and it likely reduces the infectivity of newly produced virions. BBR and OLX are effective in a physiologically relevant cell culture model at low micromolar concentrations and it could be considered for further assessment.

## Data Availability

The data generated in this study are included in the publication and its Supplementary Materials.

## Supplementary Materials

The following are available online at https://www.mdpi.com/1999-4915/13/2/282/s1, Figure S1: SARS-CoV-2 replication kinetics in Vero E6 cells, Figure S2: Plaque phenotype of SARS-CoV2 Nijmegen1 isolate, Figure S3: SARS-CoV-2 spike protein expression in primary nasal epithelial cells, Figure S4: Cytokine and chemokine production from SARS-CoV-2 infected primary nasal epithelial cells.
